# Epidemiological contemplation for a currently pragmatic COVID-19 health passport: a perspective

**DOI:** 10.3389/fpubh.2024.1347623

**Published:** 2024-02-13

**Authors:** Radha Ambalavanan, R Sterling Snead, Julia Marczika, Alex Malioukis

**Affiliations:** Research Department, The Self Research Institute, Broken Arrow, OK, United States

**Keywords:** SARS-CoV-2, COVID-19, health passport, health certification, COVID-19 status certification, digital health passport, epidemiological challenges

## Abstract

The coronavirus disease 2019 (COVID-19) has caused a global pandemic that has wreaked havoc on the lives of millions of people around the world. Confinement measures aim to reduce the epidemic's spread and minimize the burden of morbidity and mortality. In response to the challenges caused by the pandemic, digital health passports have been developed exponentially. We highlight the latent epidemiological barriers to health passports to achieve standardized digital care platforms. This review paper not only highlights the epidemiological barriers but also articulates the possible infrastructure required to make the International Standard for a multi-factor authenticated and validated health passport.

## 1 Introduction

At the start of 2020, a novel coronavirus, severe acute respiratory syndrome coronavirus 2 (SARS-CoV-2) belonging to the *Coronaviridae* family arose and led to coronavirus disease 2019 (COVID-19). This virus was first identified in Wuhan, China and has since spread worldwide. As of November 2022, more than 600 million cases of COVID-19 have been reported, with more than 6.5 million deaths (1–3).

The impact of the COVID-19 pandemic on mortality and morbidity is dramatically increasing over time. The consequences of COVID-19 are catastrophic, as the lockdown measures to contain the spread of the virus not only crippled the economy but also curtailed civil liberties and confined people to their homes. The devastating impacts of the COVID-19 pandemic accelerated the research, production, and distribution of new vaccinations, which occurred at a rate extraordinary in the history of humanity. Currently, millions of people around the world are being vaccinated against COVID-19; as of November 2022, more than 12 billion (12,885,748,541) vaccine doses had been administered (4, 5). It is believed that this vaccination measure will pave the way for economic recovery, restoration of people's social life, physical and mental well-being, and the reinstitution of freedoms. As a result, governments throughout the world have been looking into the prospect of health passports to allow more freedom of movement both within their countries and internationally (6–8).

### 1.1 Health passport for the COVID-19 vaccine to safely resume activities

According to the literature analysis by Karopoulos et al. (9) on the COVID-19 digital certificate, a significant number of countries have devised solutions that are either paper based or digital, validated by electronic means, and support at least one kind of vaccination proof, diagnostic test, or immunity certificate, as follows: (i) vaccination certificates, which state whether an individual is vaccinated or not; (ii) diagnostic test certificates, which report whether an individual has undergone a specific type of testing; and (iii) immunity certificates, also termed immunity passports, which authenticate an individual's past infection status and the development of antibodies. Digital health passes could become an important vector for post-pandemic life and prevention for subsequent pandemics (10, 11).

### 1.2 Domains for implementation and evaluation of health passports

The World Health Organization (WHO), the International Air Traffic Association (IATA), and the World Economic Forum have explored possible standards and mechanisms for implementing immunity passport solutions, which reflects the probability of their initial implementation being for international travel (12). In the COVID-19 pandemic setting, health passports are envisioned for these sectors: (i) international travel, (ii) returning to work (e.g., healthcare workers, teachers, people of the transportation crew, workers at ports of entry), (iii) education (e.g., academic institutions), (iv) attending athletic events, (v) attending mass gatherings, (vi) immigration, (vii) government agencies (which may include front-line workers, health department representatives, hospital staff), and (vii) government policy stakeholders (13). A vaccine passport should address specific issues based on each country's needs, logistics, and epidemiological determinants.

The main objective of this review is to contemplate the many epidemiological variables in successfully introducing health passports on a large scale. Apart from the assumed list of epidemiological barriers, our review highlights the infrastructure required to operationalize idea health passport and successfully overcome the inherent challenges.

To accomplish this, we conducted a thorough literature analysis from January 2020 to January 2023, utilizing PubMed, Medline, Google, Scopus, Google Scholar, and WHO websites. Our English language searches focused on epidemiological factors, testing barriers, immunity, vaccination, variants, data and research gaps, and COVID-19 health certificate validation. We used several keywords, including “COVID-19 passports”, “digital health passport”, “health certification”, “vaccination passports”, “vaccine verification”, “vaccination campaigns”, “testing requirements”, “privacy and security”, “health information exchange”, “challenges”, as well as “obstacles.” The use of Boolean operators and snowballing approaches yielded 135 related articles. The study addresses identified gaps and presents a comprehensive overview of considerations for implementing COVID-19 health passports.

## 2 Scenarios for Covid-19 epidemiological variables—intrinsic overview

For crucial information on the spread of the pandemic, it is important to infer the transmission dynamics of COVID-19 in a variety of contexts, particularly in geographic areas with poor access to healthcare, dense populations, and a high prevalence of other neglected regional diseases (14). Heterogeneity remains broad in the modes of transmission and viral shedding, primarily through respiratory droplets, aerosols, fomites, other body fluids, and secretions, throughout the infectious period and including pediatric and asymptomatic infections as well. Highly infectious individuals shed tens to thousands of SARS-CoV-2 virions per minute through droplets and aerosols while breathing, talking, and singing (15–17).

### 2.1 Global strategies for disease containment through non-pharmaceutical interventions

**Figure 1:**
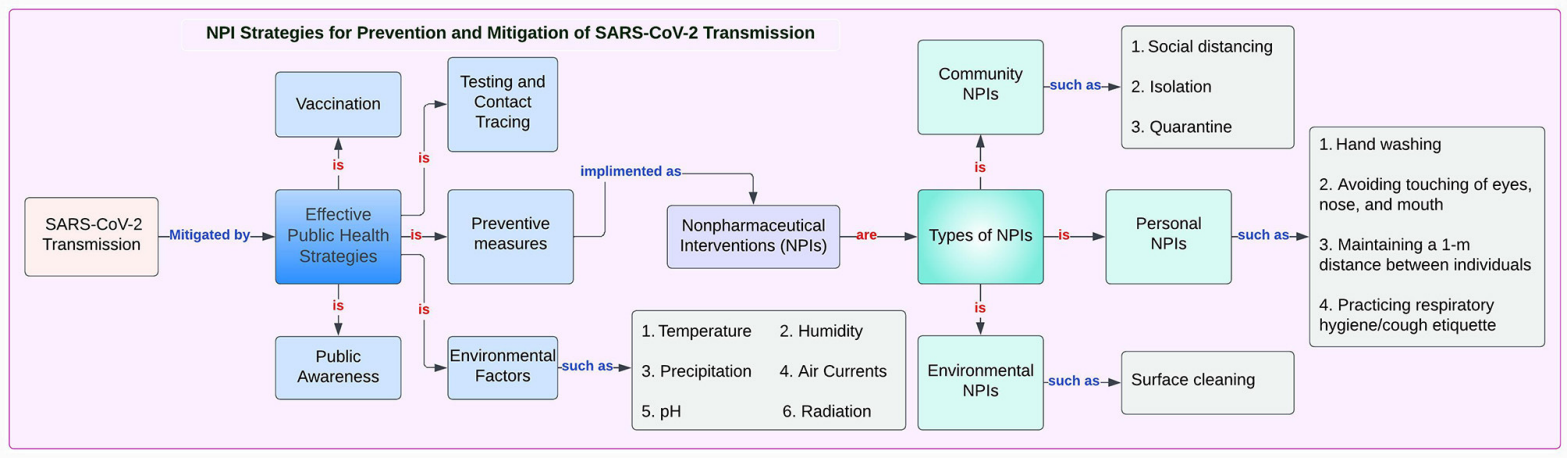
NPI Strategies for Prevention and Mitigation of SARS-CoV-2 Transmission.

From the beginning of the pandemic to this moment, public health counter measures have involved the use of already existing interventions to limit the spread of the virus (Figure 1) (18–23).

### 2.2 New variants change the herd-immunity equation

The race to vaccinate the world against COVID-19 is already facing significant challenges, with distribution and allocation issues compounding the problem. The new virus variants are adding to the woes, especially because they are more transmissible and resistant to vaccines. According to immunologist Ester Sabino at the University of São Paulo, Brazil, and her colleagues, more than 60% of individuals with COVID-19 had been infected by June 2020, a rate more than sufficient to achieve herd immunity. However, in January 2021, a massive resurgence in the number of cases occurred, and this spike happened because of the emergence of a new SARS-CoV-2 variant, P.1., which undoubtedly shows that previous infection and immunity never provided any protection.

Ferrari, an epidemiologist at Pennsylvania State University Center for Infectious Disease Dynamics, states that higher immunity rates could increase pressure on sustainable herd immunity favoring variants that can infect already immunized people. Therefore, there is an excellent reason to build and infrastructure to monitor novel variants in the setting of vaccination that can produce new variants in response to evolutionary pressure (24).

A potential framework was developed for identifying and estimating community-wide immunity to COVID-19 using data reportable to local public health authorities. However, biological factors and changing behavioral contributors, as well as context-specific factors make it hard to determine specific geographical thresholds for herd immunity (25).

### 2.3 Significance of knowing the incubation period and clinical features together with disease severity

During an outbreak, knowing the incubation period of an infectious illness—defined as the time between exposure to the causative agent and the symptom onset can provide crucial information, such as when infected persons will be symptomatic and are most likely to spread the disease. Due to the fact that the symptom onset reflects the pathogen growth, replication rate, and toxin excretion, the incubation period provides insight into the etiology and origin of a disease when these elements are unknown, leading to potential treatment strategies. Active monitoring during the incubation period requires exposed persons to report their status to local health authorities on a daily basis (26, 27).

In a scoping review of the literature, Zaki and Mohamed (28) report that the average incubation period for the virus is around 7.8 days, whereas WHO and European Center for Disease Prevention and Control (ECDC) reported an incubation period of 0, 14 days and 212 days, respectively. Infection with COVID-19 can occur in three stages: (i) an early infection, marked by a viral response; (ii) a pulmonary phase; and, finally, (iii) a hyper-inflammation phase, marked by an inflammatory response from the host.

The early stage of infection is generally associated with fever, a dry cough, and mild constitutional symptoms. The pulmonary phase involves shortness of breath with or without hypoxia. The hyper-inflammation phase involves acute respiratory distress syndrome, shock, and cardiac failure (29).

Clinical symptoms of COVID-19 are similar to those of the common cold; remarkably, however, the fatality rate with COVID-19 remains at 2%−3% for individuals with either health complications or previous comorbidities, and especially among older adults. The specific comorbidities include cardiovascular disease, diabetes mellitus, chronic respiratory disease, hypertension, and cancer; notably, lifestyle factors such as smoking and obesity are associated with adverse outcomes (30). Acute respiratory distress syndrome is the primary complication in patients with severe illness and may develop shortly after the onset of shortness of breath. Other complications include arrhythmias, acute cardiac injury, and shock. Thromboembolic complications, including pulmonary embolism and acute stroke, have also been reported. Some patients had laboratory evidence of an enduring inflammatory response that was associated with critical and fatal outcomes.

The SARS-CoV-2-related morbidity and mortality are considerably lower among young children and adolescents, and children may be less vulnerable to infection (31). Although asymptomatic infections have not been systematically studied, some studies estimate that approximately 20%−50% of infections are asymptomatic, with significantly higher rates of asymptomatic infections among children (32).

### 2.4 COVID-19 vaccination as a pathway for trustworthy protection

Numerous vaccines were developed worldwide against SARS-CoV-2 and approved by various countries (33). Table 1 summarizes types and descriptive overview of COVID-19 vaccines. This structured overview enhances understanding and facilitates essential features of the current worldwide immunization schemes.

**Table 1:** COVID-19 vaccines: types and descriptive overview.

**Category**	**Description**
Genetic vaccines	Utilize SARS-CoV-2 specific DNA/RNA sequences to stimulate an immune response.
Viral vector vaccines	Use alternative viruses as carriers for SARS-CoV-2 genes.
Whole virus vaccines	Based on the presence of an inactivated form of the virus.
Protein-based vaccines	Incorporate select virus spike proteins.
Repurposed vaccine	The Bacillus Calmette-Guérin vaccine is repurposed to stimulate the immune system.

However, significant skepticism persists around COVID-19 vaccines regarding their safety, long-term adverse effects, insufficient testing and clinical trials, and the violent pro-inflammatory response from T-cells. Various factors including insufficient neutralizing antibodies, weak memory T-cells responses, and new SARS-CoV-2 variants, may contribute to low efficacy and impact long-term protection from infection (33).

### 2.5 Tackling the threatening SARS-CoV-2 variants: an era of scientific challenges

**Figure 2:**
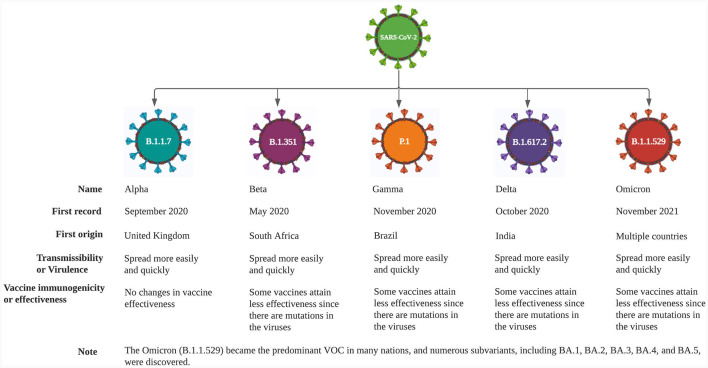
Variants of concern as of December 11, 2022.

The global emergence of several SARS-CoV-2 variant strains has resulted in an international population that is susceptible to infection, increased disease severity, seasonality of dissemination, transmissibility, and different modes of transmission, all of which are undoubtedly a significant threat to the control of the COVID-19 pandemic, as well as causing a vital public health burden. Together, these factors contribute to substantial morbidity, mortality, and concomitant economic losses worldwide, dramatically increasing over time (5, 34). The SARS-CoV-2 variants are classified by WHO into three categories: (i) a variant of concern (VOC), (ii) a variant of interest (VOI), and (iii) a variant of high consequence (VOHC). As of December 11, 2022, WHO has designated five VOCs: alpha, beta, gamma, delta and omicron (Figure 2) (35–39).

The increasing spread of COVID-19 variants has resulted in a substantial increase in the number of individuals experiencing prolonged symptoms resulting in long COVID, posing challenges for patients, their families, and the economy. In addition, this situation could put a strain on healthcare systems and have an impact on global workforce efficiency. The key to overcoming these epidemiological barriers requires providing sufficient resources, intended research, and comprehensive support services along with technological innovation. By achieving the diverse needs of long COVID patients and reducing the burden on individuals and healthcare systems, we can effectively address these challenges (40).

### 2.6 Unified approach for air travel recommendations

According to a narrative review by Bielecki et al. (41), the temperature screening method to detect COVID-19 infection is highly ineffective, particularly regarding the lack of benefit of this approach to identify infected young people. Future strategies at airports could include the following: a telemedicine approach, performing systematic rapid tests, performing a combination of saliva and antigen tests, and ensuring that travelers complete self-assessment forms before flights. Henceforth, saliva testing on arrival could be used to isolate and quarantine the traveler, which would ultimately help reduce the number of quarantines. Another approach is that, from the moment that travelers enter the airport and until they leave the airport, they must practice proper hand hygiene and physical distancing. Always covering the face is one of the key elements for preventing SARS-CoV-2 transmission. The passengers within two rows of an index case are at higher risk, despite the high-efficiency filtering used in aircraft. Even with one positive case aboard, however, the findings of a retrospective study must be considered, which showed that the transmission of SARS-CoV-2 is one case for every 27 million travelers, with stringent in-flight hygiene measures. Most flight crews disinfect the aircraft and enforce wearing face masks/shields and following social distancing to a certain degree; however, their guidelines are indeed challenging to research, and other precautions must be more transparent and less confusing to interpret. Non-pharmaceutical interventions such as masks and the use of hand sanitizers are mostly recommended from door-to-door and during travel. The IATA guidelines are straightforward. However, there is no common platform for contact tracing and telemedicine approaches using preflight questionnaires and COVID-19 test results. It is now necessary for all stakeholders to take a unified approach, to validate existing rapid tests, and to form an expert committee to make prevention strategies more systematic so that evidence-based air travel recommendations are followed (41, 42).

The introduction of vaccination passports does not withdraw any recommendations for wearing masks and social distancing on flights. Whether digital or paper-based, the evolution of vaccination passports is certain; however, this outcome requires careful navigation (43). Indeed, the contemporary echo chamber effect may instigate vaccine hesitancy and unethical practices; one of the most likely weaknesses is that people could falsify vaccination records. Regardless, one principle is certain: It is important for each person to actively contribute to the ethical protection of public health, the economy, and society as a whole.

## 3 Epidemiological obstacles renders impact on the verification of variables in the health passport—elaborated review and possible resolutions

Organizations worldwide collaborating to develop plans for further opening up the economy and restoring the perception of normalcy around the world have achieved significant results. The economy has made substantial progress in its path to recovery, with air travel almost reaching pre-pandemic levels. It is important to acknowledge these positive advancements. Vaccine passports, travel passes, and global health certificates are all different names for the same purpose. During the pandemic and pre-pandemic periods, a significant reduction in air travel was apparent, with a decrease in the number of passengers departing. Global travel trends are essential to understand in this context, and they indicate that the fastest and most resilient travel flows for recovery are domestic flights and travel to neighboring countries, last-minute travel, visits to friends and family, and quintessential travel.

### 3.1 Potential epidemiological barriers and stumbling blocks for verification and validation of the health passport

The following are the five essential elements for recovery and policy changes for the introduction of vaccination passports: (i) competition, (ii) epidemiology, (iii) technology, (iv) ethics, and (v) politics—neglecting to take these factors into account could result in unforeseen distress in the future. Health passports have the potential to protect the air travelers, and residents at the destination and transit stops. Both the IATA and WHO, various countries, and continental agglomerations jointly handle the implementation of health passports. The essential data to be included in a health passport are the following: (i) date of passport issue, (ii) place of passport issue, (iii) type of vaccine, (iv) information on the passport verification process, and v) validity of the passport.

The health passport program can be successfully realized only when the minimum scale is exceeded, considering the views of epidemiologists on a global scale. On the contrary, without sufficient scale, the health passport program can have the opposite effect of the original intention, leading to “bubbles” instead of re-connecting the world. These “bubbles” will essentially align with the current political, ideological, and economic considerations (44). Moreover, several challenges are related to epidemiological research, similar to the challenges posed by the pandemic and variants, in terms of implementing health passports. We categorize these epidemiological barriers into four domains (i) testing, (ii) immunity, (iii) vaccination, and (iv) variants, all of which play an essential role in implementing health passports.

### 3.2 Uncertainties of current COVID-19 testing and diagnostic strategies

At the moment, two types of viral tests can be used: nucleic acid amplification tests (NAATs) and antigen tests. Antibody testing, also known as serology testing, may tell if the individual has had a prior infection. Antibody testing must not be used to diagnose a present infection (45). Ribeiro da Silva et al. (46) pointed out that laboratory diagnosis is “crucial for the clinical management of patients and the implementation of disease control strategies to contain SARS-CoV-2 at the clinical and population level.” Despite the fact that the current testing strategies have proven to be the golden standard, many uncertainties around the testing process might impact freedom from the pandemic and the health passport.

#### 3.2.1. Falsification of a negative polymerase chain reaction test

A person who produces significantly higher amounts of infectious aerosols may be more likely to spread the infection and be accountable for the “super spreader effect,” in which that person is responsible for the infection of an unusually large number of susceptible persons (47). Likewise, the WHO scientific summaries indicated that persons with a negative test and none of the symptoms are less likely to cough and sneeze, making them probably in charge of most transmissions (48). Therefore, the current negative PCR test in the individual and transmission from people without COVID-19 symptoms are the major problems.

The decision-analytic model assessed by Johansson et al. (49) reported various levels of transmission of SARS-CoV-2 from pre-symptomatic, defined as infectious before symptom onset, never symptomatic, and symptomatic individuals across a range of scenarios in which the proportion of transmission from people who never develop symptoms (i.e., those who remain asymptomatic) and the infectious period varied according to published best estimates. This degree of variation means that the outcomes may not be valid for an extended period of time.

#### 3.2.2 Contingencies around a positive antibody test

A positive antibody test suggests that individuals may have antibodies from a previous infection or vaccine for the virus that causes COVID-19 (50). The accuracy of the test result depends on the test being used and the prevalence of SARS-CoV-2 immunity in the population (51). If the test is performed between 15 and 35 days after symptoms appear, or more than 1 week after infection, a positive result may be caused by the existing infection and can thus imply a current risk of transmission (52). The production of antibodies after the infection seems to be variable in terms of amount and duration. The duration of protection is unclear, and protection may decrease over time (53). Still, the evidence for the efficacy of immune antibodies is insufficient to effectively ensure the accuracy of health passports.

#### 3.2.3 A chaotic state against the negative rapid antigen test

Rapid antigen tests are helpful to determine if an individual has COVID-19 before exposure to the crowd or event or if symptom onset has occurred. The rapid antigen SARS- CoV-2 tests and some rapid NAATs are considerably less sensitive than most real-time-polymerase chain reaction (RT-PCR)-based NAATs. The variation in the performance among different tests is substantial (54). Special attention is required regarding the accuracy of different tests regarding false-negative results, most notably when non-professionals rather than laboratory scientists administer the tests (55, 56). Dauntingly, a high rate of false-negative results makes it harder to control hospital infections and make clinical decisions (57). Apart from these concerns, the passengers who carry false COVID-19 test results and false health passports to travel might jeopardize the sector's efforts to limit the spread of the disease and especially as air travel has reopened completely to pre-pandemic level.

#### 3.2.4 Dilemmas in testing: positive RT-PCR test results in patients recovered from COVID-19

According to a review by Lan et al. (58) a proportion of cured patients may continue to carry the virus. Notwithstanding, a follow-up study by Wu et al. (59) suggested that recovered patients with repeat positive PCR tests were not infectious when the test was performed. The duration of immunity from disease is unclear and appears to be variable (60). A symptomatic infection is typical, and it is rarely missed by various tests (61). Most likely, these conflicting results could affect the usefulness of a health passport.

#### 3.2.5 Noteworthy resolutions for uncertainties in COVID-19 testing and diagnostics

Health passports built based on antibody testing or tests for infection confront significant technical, legal, and ethical challenges (62). Today, the existing scientific reviews provide possible solutions. However, well-designed experiments are always necessary for finding a solution to diagnostic and testing uncertainties.

##### 3.2.5.1 Test sensitivity and specificity

Grassly et al. (62) suggest that molecular tests must have high specificity to avoid false negative results because a lower specificity would reduce the usefulness of a molecular-based health passport. To maximize financial grant approval for the fight against COVID-19 and the implementation of health passports, it is now time to invest in testing capacity, policies, and planning (63).

##### 3.2.5.2 Sample pooling

Pool testing strategies combine samples from specimens (e.g., throat swabs) from numerous people and test them as a group in a single test. To elucidate these strategies, Bish et al. (64) implemented a robust pooling strategy within a sequential framework, which shared pool sizes weekly for each risk group based on test data from the previous week. As demonstrated by this study, a robust approach for pooled testing can be a helpful strategy for significantly increasing the COVID-19 screening and testing capacity. Due to the dynamic and uncertain prevalence of the disease, this customization is beneficial for testing various populations.

##### 3.2.5.3 Quality control of COVID-19 testing

Infection control measures, outbreak monitoring, and management are mostly based on test results. Hence, quality control at all levels, from design up until end use, as well as high internal standards, is Health Kit identifiers needed to obtain an acceptable report in diagnostic testing and the implementation of new tests.

Significantly, obtaining official, formal FDA approval, optimization of additional tests, and better extended clinical and epidemiological validation are also required. Moreover, biobanks and actual patient monitoring continue to be lacking, and artificial intelligence and machine learning tools must be developed and implemented in data interpretation (63). We expect that these testing-related interventions will be restorative and help retain accurate information in the health passport.

### 3.3 The unpredictability of immunity against COVID-19

The immune system of more than 95% of individuals recovering from COVID-19 showed long-term memories of the virus up to 8 months after infection. In the same way, clinical research suggests that people who are vaccinated against SARS-CoV-2 will develop similar long-term immune memories (65).

#### 3.3.1 Skepticism about lasting immunity to COVID-19

Many questions remain about natural immunity and SARS-CoV-2 vaccine induced immunity. According to Baraniuk's (66) reviews, in some cases it is unclear how long the developed immunity will persist when the body's immune system responds to COVID-19 infection. Notably, COVID-19 is an entirely new disease, and scientists are still working specifically on how the body repels it. Although it is difficult to state definitively, one could predict that the immunity could last for several months or up to 2 years based on what is known about other viruses and what has been seen to date in terms of antibodies in COVID-19 patients and individuals who have been vaccinated. Moreover, concerning immunological studies on COVID-19, outcomes are inconsistent, so it is not easy to develop a “ballpark figure” estimate of lasting immunity.

#### 3.3.2 Suspicion of perceived personal immunity

The use of vaccination passports may suffer severe problems if the underlying degree of immunity is not understood and with limited knowledge of the actual risks for seen. The dynamics of humoral immune responses after SARS-CoV-2 infection and the certainty of the potential for reinfection would require decades to understand, so these passports will be deployed with little understanding of the real risks (67).

#### 3.3.3 Imprecision about antibodies and immunity in COVID- 19 infected persons

According to Wang et al. (68), research demonstrates that after a SARS-CoV-2 infection, most people, even those with mild infections, seem to have some protection from the virus for at least 1 year. In addition, other research confirms that vaccinating these individuals considerably improves their immune response and gives them strong resistance against the variants of concern, including the delta variant (B.1.617.2) (69).

A serological-based study conducted by Ripperger et al. (70) demonstrated that individuals who recovered from COVID-19 had observed antibodies still remaining in their blood 5–7 months after the illness. Evidence suggests that neutralizing antibodies last several months for individuals already infected with COVID-19 but then gradually decrease over time. Additional investigation is required to determine precisely how the body fights SARS-CoV-2 and how long multifunctional antibodies could play a defensive role after infection or vaccination. This knowledge is essential to grant access to health passports.

#### 3.3.4 Noteworthy resolutions for unpredictability in immunity against COVID-19

Being able to represent “active immunity” is essential in developing protocols to protect the population globally and to cure resistant diseases in the future. Accordingly, the available studies to date suggest that determining the exact duration of immunity is unattainable by the scientific goals of the researchers (71). Marovich et al. (72) reported that neutralizing monoclonal antibodies to SARS-CoV-2 is beneficial for therapeutic and prophylactic applications. According to their suggestion, monoclonal antibodies are an additional method for preventing COVID-19. A passive infusion of monoclonal antibodies as pre-exposure or post-exposure prophylaxis could immediately protect against infection, which could last for weeks or months. More recent technologies modify the fragment crystallizable region of the antibody to extend the half-life of the monoclonal antibodies and may supply potentially protective levels for months, based on the requirements for monoclonal antibody concentration. In the event of an outbreak, it may be beneficial to administer monoclonal antibodies to nursing home residents to mitigate disease progression during the early stages of rapid infection that may go undetected.

According to Rafi Ahmed, a viral immunologist at Emory University in Atlanta, Georgia, SARS-CoV-2, like all pathogens, uses several mechanisms to disable and escape the host immune response. This mechanism allows the virus to survive better by causing the host's innate immune response to be inefficient. It is challenging to dissect how much collateral damage is caused by the virus itself and what percentage is the immune response. Because of these uncertainties, scientists will develop a new method, nearing a combination therapy (73).

The Kirkcaldy et al. (74) viewpoint study conveyed that amid this uncertain public health crisis, thoughtful and robust scientific data are essential to provide guidance on public health policy, planning, and practice. According to existing reviews, to improve the outcomes of COVID-19 studies, several governmental interventions, including direct financial investments, loans, and policymakers, must be pursued to allow scientific innovation teams to equip the necessary facilities and test their new ideas. It is our hope that these movements will support creating the health passport more efficiently.

### 3.4 Perplexities about COVID-19 vaccination

Vaccinations save millions of lives annually. Although not without its repercussions, numerous safe and effective vaccines prevent people from becoming critically ill or dying from COVID-19.

#### 3.4.1 The right vaccination strategy

As WHO has stated on numerous occasions, the pandemic cannot end until the entire world is vaccinated. The choice of the right vaccination strategy is an epidemiological challenge, with so many different approaches taken worldwide (44). The government must provide insights to the rural, frontier, and tribal organizations about the COVID-19 vaccine roll-out. It is considered vital to discuss challenges in rural communities, including access barriers and vaccine hesitancy, and to propose innovative strategies to address these challenges. This approach will help determine unmet needs and potential strategies as the vaccination process moves forward.

#### 3.4.2 Protection against infection and transmission after vaccination

While it will be essential to have COVID-19 vaccines that prevent infection and transmission, proof is still needed that protection is happening. Protection against transmission can be hard to prove because several factors can cause a decline in infections (75). Although it would be unusual for vaccines not to prevent infection, the level of protection is unknown, impacting the success of implementing the health passport.

#### 3.4.3 Correlates of protection against symptomatic and asymptomatic infections after vaccination

The messenger RNA (mRNA) vaccines produced by Pfizer-BioNTech (BNT162b2) and Moderna (mRNA-1273) are highly effective in stopping SARS-CoV-2 infection in real-world conditions. Based on previous clinical trials, these vaccines were known to be effective in preventing symptomatic diseases (76). What was not known, however, was whether these vaccines were stopping asymptomatic infection. The Centers for Disease Prevention and Control and Prevention (CDC) conducted a prospective cohort study in eight locations across the United States during the period December 14, 2020, to March 13, 2021. The organization confirmed that prospective cohorts of 3,950 healthcare personnel, first responders, and other essential and front-line workers completed weekly SARS-CoV-2 testing for 13 consecutive weeks. The CDC reported that receiving two doses of an mRNA vaccine provides 90% protection from infection. Even one dose is considerably effective, lowering infection rates by approximately 80%. These findings indicate that authorized mRNA vaccines confer more long- lasting protection against severe outcomes of hospitalization and death than against asymptomatic and symptomatic SARS-CoV-2 infections (77). However, supplementary data are needed to specify a percentage of protection to analyze the risk factors and support the most effective health passport.

#### 3.4.4 Individuals receiving just one dose, mix and match vaccines

The clinical trials of the Pfizer and Moderna vaccines were conducted with two vaccinations separated by either 21 or 28 days. In people who have already had COVID-19, a single dose of the vaccine produces a robust antibody response similar to a second dose (78). However, at present, there is no single-dose recommendation for those who have already been infected, and the recommendation to complete the vaccine series is primarily due to the concern about the emergence of variants. Until further data are available, all individuals receiving available mRNA vaccines should be given two doses. As of October 20, 2021, the CDC and FDA authorize the use of heterologous booster doses (or “mix and match”) for currently available COVID-19 vaccines in the United States (79). It is important to consider the risks and outcomes of mixing coronavirus vaccines to develop a holistic health passport.

#### 3.4.5 Timing of COVID-19 vaccine booster doses: the necessity for boosters and the use of monovalent and bivalent boosters

A growing number of infections are caused by the highly contagious variants of SARS-CoV-2 and indicate that COVID-19 vaccine-induced immunity could diminish over time. Some countries are looking at the possibility of giving other doses to those who have been completely vaccinated. However, scientists do not know if most people need these booster doses (80, 81).

The antigenic evolution of the SARS-CoV-2 virus yet to be researched will answer most of the questions about booster vaccination. For example, repeated vaccination for influenza is required, whereas other vaccinations for infections, such as measles, are provided during childhood and protected for life. As a result, many questions arise regarding the lasting immune protection, the nature of that protection, protection against the likelihood of reinfection, and the healthcare disease burden that the system can tolerate (82).

Following the FDA regulatory action on August 31, 2022, the EUA for the bivalent formulations of the Moderna COVID-19 vaccine and the Pfizer-BioNTech vaccine were amended for use as booster doses after 2 months of either primary or booster vaccination. Subsequently, CDC updated its recommendations following the FDA amendments for COVID-19 boosters for people aged 12 years and older from Pfizer-BioNTech and for those aged 18 years and older to provide better protection against the recently circulating COVID-19 variants (83).

Vaccine-induced protection likely depends on variables such as the vaccine product, primary vaccination schedule, vaccine recipient's age and medical conditions, exposure risk, and the specific variants in circulation. Thus, the decision to recommend a booster vaccine depends on a complex set of variables beyond consideration of clinical and epidemiological data alone.

The following are some of the markers to be considered: (i) epidemiology and burden of disease; (ii) assessing the performance of booster doses; (iii) optimal timing of the booster dose; (iv) consideration of homologous vs. heterologous boosters; (v) possibility of dose-sparing for booster doses; and (vi) booster needs of individuals already infected (84).

#### 3.4.6 Vaccine efficacy for pregnant and lactating women

Craig et al. (85), addressed the considerations required for COVID-19 vaccination during pregnancy. Key findings included: (i) COVID-19 infection among pregnant women has been linked to an increased risk of morbidity and mortality; (ii) a significant proportion of healthcare workers are pregnant and will potentially be eligible to be vaccinated before studies can be conducted during pregnancy; and (iii) FDA-approved vaccines must not be withheld from women solely based on their pregnancy or lactation status when they otherwise meet the vaccination criteria. In a systematic review and meta-analysis study on the effects of COVID-19 immunization in pregnancy, Prasad et al. (86) found that COVID-19 mRNA immunization during pregnancy appears to be safe and is linked to a decrease in stillbirth. According to CDC reports on breastfeeding mothers, those who had received mRNA COVID-19 vaccines protected their infants through the antibodies in their breastmilk, with no evidence showing any harmful effects on either the infant or the mother (87). When providing epidemiologically validated health passports, additional data are required to decide what level of protection these antibodies provide to pregnant women and infants of lactating mothers.

#### 3.4.7 Jabs for infants and children under EUA

Children are more susceptible to having asymptomatic cases of COVID-19 and could act as unknown carriers of SARS-CoV-2 (88). According to Ludvigsson et al. (89) a systematic literature review revealed that children linked to exposure and host factors are the largest age group of asymptomatic carriers of SARS-CoV-2, followed by adults and older adults. It is well-known that a child's immune system is not well- developed, and the maturity and binding capacity of ACE2 in children may be lower than that of young adults. On October 29, 2021, the FDA authorized and the CDC recommended that children age 5–11 years could receive an age appropriate dose of the Pfizer-BioNTech COVID-19 vaccine for emergency use to help protect against infection (90). According to the FDA, the bivalent approval for the Pfizer-BioNTech COVID-19 Vaccine in children aged 5–11 years stands as valid in addition to the earlier approval for a monovalent vaccine (91). However, it would be hard to justify a decision to require children to receive a vaccine because the role of children in the spread of infection to adults and to those at risk remains questionable (92).

#### 3.4.8 Safety for people after they are completely vaccinated

Unestablished facts about the duration of vaccine-induced immunity and the risk of new variants with complete vaccine-escape capabilities raise challenges about the validity period of health passports and ensuring that holders of health passports are still immune to circulating viral strains (93).

#### 3.4.9 Noteworthy resolutions for perplexities in COVID-19 vaccination

New biomarkers are essential to manage patients by facilitating early diagnosis of severe COVID- 19 and play a vital role in developing a COVID-19 vaccine. Use of these biomarkers can speed clinical trials, reduce costs, guide participant selection, reduce patient safety, and enable easier verification of the mechanism of action (63). Thus, biomarkers are a relevant factor in developing a COVID-19 vaccine. Efficient vaccine effectiveness studies are needed to nourish greater immunogenicity and to guide periodic revaccination of the general population. The opinion article by Baay and Neels (94) represents that the controlled human infection (CHI) model could help speed vaccine development. To support vaccine research, CHI can provide fundamental security, tolerability, immunogenicity, and efficacy.

Compatible collaboration is necessary to develop medicines and vaccines. Optimally, information should be shared among the currently available digital technologies, regional and international health surveillance institutes, industrial partners, and innovation drivers such as bioinformatics data management (termed big data), biobanks, and innovation science teams (63).

Therefore, each vaccine category must be evaluated separately to provide essential scientific information for the COVID-19 health passport. In this state of affairs, crucial scientific information such as the duration of immunity and efficiency in reducing infection and virus transmission must be examined (95). Correspondingly, WHO recommends a preference for standardized study reporting based on the Strengthening the Reporting of Observational Studies in Epidemiology (STROBE) guidance. The STROBE statement aims to assist authors in enhancing the reporting of observational studies and facilitating critical evaluation and understanding of the results (96). In accordance with the viewpoint study of Gostin et al. (10) ideally the digital health passports would include the completion dates of the vaccine series to determine the expiration date once the duration of the protection is more clearly illustrated. Modern evaluation studies and global scientific partnerships could be helpful in obtaining better defined details regarding vaccine protection, encouraging health passport use, and making the use of a health passport worthwhile.

### 3.5 Emergence of COVID-19 variants may hamper the freedom from infection

Scientists are steadily monitoring the new genetic changes that COVID-19 is undergoing. Some emerging variants are alarming, whereas many variants are inconsequential. The most challenging task is recognizing, tracing, and controlling those variants that may be significant.

#### 3.5.1 COVID-19 variants as game changers

Currently, the future of COVID-19 is decided by its mutations. As a natural process, often mutation does not affect the virus and may even cause disease in some cases. Variants of concern pose distress and represent a significant number of infections worldwide with high transmission rates. The foundations for a thorough understanding of why vaccination against COVID-19 is required include the level of immunity to the virus, efficacy of current vaccines against emerging variations, and international air travel that may spread the variants globally. These intense challenges can lead to a completely chaotic system of competing variants, competing vaccines, and competing passports prevails (43, 44). Protection from emerging variants of SARS-CoV-2 continues to be unclear. The Delta and Omicron variants create new uncertainty and thus lead to new revisions of the health passport.

#### 3.5.2 Vaccine effectiveness against new variants

New variants will continue to emerge, and it is important to understand the phenotypes of emerging variants in terms of infectious disease, transmissibility, virulence, and antigenicity. It is also essential to quantify the phenotypic effect of specific mutations present in the variants, both individually and in combination with other mutations (97). When the Omicron variant evolved in early 2022, persons with healthy immune systems who were eligible to receive the third and fourth doses of the COVID-19 vaccination were provided significant protection, according to a recent Morbidity and Mortality Weekly Report.

According to the CDC, experts reviewed VISION Network data for more than 214,000 emergency department/urgent care visits and more than 58,000 hospitalizations with a diagnosis of COVID-19-like illness in 10 States from mid-December 2021 to mid-June 2022 to assess the efficacy of 2, 3, and 4 doses of mRNA COVID-19 vaccines (Pfizer-BioNTech or Moderna) among adults with healthy immune systems (Table 2) (98, 99). Indeed, it is unclear to what extent the results of an infection might be attributed to prejudice because of test-seeking performance being affected by vaccination status.

**Table 2:** Effectiveness of booster vaccines against new variants.

**Occasions/incidents**	**Vaccine effectiveness (VE) before COVID-19 booster**	**Vaccine effectiveness (VE) after COVID-19 booster**
When BA.1 became predominant variant	VE was 61% for two doses against COVID-19 associated hospitalizations	VE increased to between 85–92% after the third/booster dose
When BA.2/BA.2.12.1 became the predominant variant	VE was 24% for two doses against COVID-19-associated hospitalizations	VE increased to between 52–69% after a third/booster dose
Emergency department and urgent care encounter attained	Attained lower VE during BA.2/BA.2.12.1 predominance	Attained higher VE with 3 or 4 doses compared to VE with 2 doses
Adults ages 50 years and older during BA.2/BA.2.12.1	VE against COVID-19–associated hospitalization was 55% higher than 4 months after a booster/third dose	VE against COVID-19–associated hospitalization was increased to 80% more than a week after the fourth dose

#### 3.5.3 Noteworthy facts: learning from the management of similar deadly viruses in the past

The influenza A (H1N1) virus caused the 1918 flu pandemic; the 1957 flu pandemic was caused by an influenza A/H2N2 virus; and the 1968 flu pandemic was caused by influenza A/H3N2 virus; moreover, the 2009 swine flu pandemic was caused by the H1N1 virus. The pattern shows declining fatalities year over time due to vaccination and exposure to pathogens for natural immunity (82, 100). Based on this pattern, booster doses for the SARS-CoV-2 virus are likely to be required at specified intervals until proper drugs and therapeutics are developed.

### 3.6 Concerns regarding potential vectors and animal reservoirs for disease eradication, as well as future prospects

The future reality of SARS-CoV-2 will also depend on its ability to become established in a wild animal population. A few diseases that have been brought under control, such as yellow fever, Ebola virus, and Chikungunya virus, persist because of animal reservoirs. It is likely that SARS-CoV-2 originated from bats and can easily infect some animals, including cats, rabbits, and hamsters, and it is particularly contagious in minks (101).

According to the hypothetical synopsis provided by Kahn et al. (102) primary vaccinations are likely to have unpredictable efficacy for subgroups of the population and it may take time to achieve herd immunity with primary vaccinations. The homogenized digital solutions must be regionally and internationally standardized in the electronic platform to document and validate COVID-19 e-vaccination certificates. Future outbreaks of a pandemic are imminent. Therefore, it is the need of the hour to develop the framework and policies guiding the integration and synchronization of digital vaccination solutions in an emergency. However, these technological interventions must follow strict ethical guidelines (103). Expectedly, until longer-term follow-up results are available, the duration of protection seems to be uncertain and thus immunity might take time to become endemic.

### 3.7 Possibility of COVID-19 becoming endemic: potential future standpoints

For both the outbreaks of SARS in 2003 and Ebola in 2014, public health measures brought them to an end. Although SARS-CoV-2 virus differs from both in comparison, possibly the current improved public health systems and successful surveillance systems can help in achieving endemic status. In contrast, however, the current pattern of human contacts, number of susceptible individuals, and transmissibility add further to the woes of putting an end to the SARS-CoV-2 virus (82, 104).

In January 2021, the journal Nature asked more than 100 immunologists, infectious disease researchers, and virologists working on the coronavirus whether it was possible to eradicate it. The prognostications from this survey revealed that many scientists expect the virus that causes COVID-19 to become endemic but that it could pose less of a danger over time. More than one-third of survey respondents believed that SARS-CoV-2 could be eliminated from some regions while it still circulates in others. In the region with zero COVID-19, a continued risk of disease outbreaks would exist, but these outbreaks could be quickly curtailed by herd immunity if most people had been vaccinated (105).

One scenario foreseen by scientists for SARS-CoV-2 is that the virus might behave similarly to the past four endemic coronaviruses OC43, 229E, NL63, and HKU1. Of these viruses, three have been circulating for more than 100 years, and two caused 15% of respiratory infections. Although the childhood immunity developed at age 6 years might wane, reinfection as an adult does not lead to any complications. Similar behaviors can be expected in SARS-CoV-2, but the results are unclear. Scientific studies have shown that immunity declines after 6–8 months and reinfection does occur. However, the body manufactures antibodies using B-cell memory and eliminates the virus using T-cell memory. This waning immunity might be the primary driver for SARS-CoV-2 to become endemic. In this endemic phase, the number of infections will be constant throughout the years, with occasional flare-ups. Achieving this state might take several years or decades, depending on how quickly herd immunity is achieved through natural infections or vaccinations (105). However, it is hard to predict when this change will occur.

## 4 Substantial resolutions for robust international health passport development: general outlook

To re-open borders without quarantine and reawaken the aviation sector, governments must be confident that they can effectively mitigate the risk of introducing COVID-19. This necessity supports the need for reliable information on the COVID-19 health status of the passengers. Notifying passengers about any necessary tests, vaccinations, and other measures they need prior to travel, providing details of where they can get tested, and providing them the opportunity to share their test and vaccination results in a verifiable, safe, and privacy protecting manner is the key to giving governments the confidence to open borders. To make it easier for passengers, key steps are to: (i) create a digital passport; (ii) ensure that the passenger tests/vaccinations meet the regulations; and (iii) share the passenger test or vaccination certificates with authorities to make travel easier (106).

The term digital health passport is a newly emerging technology, and its configuration is exclusively centered on uncertain and evolving scientific information. Some factors to consider regarding “how to use digital health passports” include whether the strategy and information can be relevant to all countries and states in all conditions. Accurate data and robust information systems are vital to refining health passports in the context of COVID-19. A multi-factor authenticated (MFA) and validated health passport could be the solution.

### 4.1 Confirmation for safe travel and integration

The effectiveness of COVID-19 vaccines is a concern with many scientific mysteries, including the inefficacy in preventing disease, use for asymptomatic infection, timing of booster doses, vaccine recipient age, population groups to be prioritized, specific contraindications, and limiting transmission, including SARS-CoV2 variants and the vaccination administration time to be determined before travel. The new COVID-19 vaccine recommendations are compiled based on the WHO Strategic Advisory Group of Experts on Immunization (SAGE) advice (107).

Clifford et al. (108) performed a study of health screening for international travel. They reported that when the number of cases are low in the exporting country, the screening may delay the onset of the epidemic by up to 1 week in the importing country. Likewise, Mandal et al. (109) conducted a mathematical modeling study. They found that if proper screening could detect 90% of asymptomatic persons, it could delay the average epidemic times by 20 days in select countries.

Because the clinical and epidemiological features of the virus are still inconsistent, assessing the potential effectiveness of the travel measures is challenging. Moreover, because of the limited transparency of the Public Health Emergencies of International Concern declaration process, the risk assessment conducted by the IHR Emergency Committee is unknown (110).

The introduction of health passports could help ensure safe travel for those carrying proof of immunization, facilitate the opening of air travel, and contribute to reviving national economies. In another regard, the reasons for ECDC or the WHO not recommending the “immunity passports” are the undefined duration and parameters of immunity, costly antibody testing, proliferating exposure to infection and reinfection issues, and new strain susceptibility.

Currently, WHO does not recommend proof of vaccination or immunity for international travel as a condition of entry. Nevertheless, WHO is working on technical specifications and standards for a smart vaccination certificate to support collaborative processes for adding the COVID-19 vaccine into the IHR updated version (43, 111). Correspondingly, the validity, the expiration date-−6 months is the current period—and the renewal of the health passport for the administered vaccination are still unanswered. Modern digital and scientific technologies, collaborations with government and private interventions, and innovation science teams can likely overcome these challenges. Thus accessing a presumably MFA and validated health passport can be within reach.

#### 4.1.1 Digital platforms as outbreak response tools

Digital solutions can be a boon in integrating care and support for people on a large scale during the COVID-19 pandemic. At any time, the vision for healthcare can be realized by focusing on flexibility and interoperability to achieve sustainability. Digital solutions can turn the idea into reality, with responsibility to ensure the best healthcare systems for people's benefit.

An effective digital platform collaborates several inter-related factors in one place, such as the following: (i) a comprehensive national epidemiologic strategy for the public health systems, (ii) interoperability of data sharing and data re-use needs to be promoted by technology and architecture models, (iii) widespread connectivity of mobile devices, and (iv) an integrated digital solution for safeguarding all stakeholders' safety and privacy following the appropriate regulatory and legislative laws (112, 113).

#### 4.1.2 Concise conceptual points for how and why to implement MFA in health passports

Traditional user identification and password logins can be easily compromised and costly to the organization. Brute-force attackers can use automated password-cracking tools until they find the right combination of usernames and passwords. Hackers have various methods to gain system access, even if a login can be locked after unsuccessful login attempts. For this reason, the MFA is used to reduce security risks.

The use of MFA can be based on the three most common categories, which usually combine the following concepts. First is something you know, or the knowledge factor. The knowledge factor requires the user to answer personal security questions. Something known is typically information such as a family member's name, birth city, phrase, and other points. Second is something you have, or the possession factor. The possession factors include a badge, security tokens, SMS (short message service, or a text message), a SIM (subscriber identity module card), and a smartphone app with an OTP (one-time password). Something you have can be a mobile phone, app, and generated code. Third is something you are, or the inherence factor. The inherence factor primarily uses biological traits, such as a retina scan, fingerprint scan, voice/face recognition, hand geometry, and digital signature. Something you are includes facial recognition, finger printing, and other biometric values. The least common factor can be the user location obtained with a global positioning system, usually provided as a built-in feature of the smartphone. For example, a bank ATM (automated teller machine) card cannot be used in the United States and then used again in Russia within a few minutes, because this incident can be identified and logically locked to prevent fraud.

Businesses today promote the 'Bring Your Own Device' approach, wherein employees are encouraged to work using their personal devices such as mobile phones and laptops, which presents a serious security risk for business. The security policy can be specified from person to person and group to group with the MFA solution.

Identity and Access Management has advanced from simple usernames and passwords to MFA, as it is now called, for which users must prove their legitimacy to authenticate and gain access to the system. The simplified MFA technique is one of the goals instead of remembering multiple passwords providing both security and fraud prevention (114, 115).

### 4.2 Data/scientific accuracy in implementing the health passport

Health passports have the potential to become a proper tool to manage COVID-19 in safer domestic, national, and international travel, although uncertainties exist on the pathway of the pandemic. Despite a unique understanding of the virus that leads to efficient disease control and vaccine development, scientific knowledge is still in progress on the effectiveness of protection offered through tests, vaccines, or antibodies, on which a vaccine passport relies.

Point-of-care testing (also called bedside testing) that shows negative evidence offers no future protection against COVID-19. With many low-accuracy tests, the reliance on test results is a challenge when implementing a passport system. The principle of a health passport is that it requires an accurate, more consistent, and reliable test system. Health modeling should support digital passports for people who work in person with vulnerable groups. Health passports normalizing individualized health risk assessment may pave the way for more widespread sharing of health data and intrusive data collection after pandemics.

The IATA develops health passports for international travel and tourism. The EU has envisaged a Digital Green Certificate, whereas WHO has developed a digital version of the International Certificate of Vaccination and Prophylaxis. A digital health passport consists of four components with different functions and purposes: (i) health information consisting of the recording and communication of vaccine status or test results through a certificate/digital certificate; (ii) identity information which may include a biometric, a passport, or a health identity number; (iii) verification to connect a user's identity to health information for checking validity; and (iv) authorization or permission, either allowing or blocking actions based on the health and identifying information.

Health passports must consider a wider breadth of the socio-technical system—one that goes beyond the scope of just data and software and includes: (i) data, (ii) software, (iii) hardware and infrastructure, (iv) people, skills, and capabilities, (v) organizations, and (vi) formal and informal institutions. Health passports form part of extensive societal systems. The public health system includes: (i) tests, (ii) trace and isolate services, (iii) mask-wearing and social distancing, or (iv) wider biometrics and digital identity ecosystem (116).

The dynamism of the health passport system should consider the differing efficacy of various vaccines, the known contrasts in efficacy with circulating variants, and changes in effectiveness over time. A health passport should be considered to work in tandem with other public health measures and cannot be regarded as a “safe haven” pass or certificate of immunity. Instead, the health passport must be considered as one of the risk mitigation tools, including NPIs. Another benefit of a health passport could be scheduling and monitoring the booster vaccines (116, 117).

Within the framework of the Razzaq (118) prospective study, a comprehensive solution for the creation of a health passport is presented, while our primary aim revolves around and goes beyond the identification of epidemiological barriers. We not only pinpoint these barriers but also offer insightful perspectives on potential solutions. This provides significant value for the design of the digital health passport for current situation, while also offering a proactive strategy for addressing future pandemics. This article demonstrates how digital health app designers in critical sectors like travel, transport, tourism, immigration, and governmental bodies can develop valuable applications from our comprehensive insights. Our goal is to establish a resilient health passport system, by providing a comprehensive analysis of the obstacles and possible remedies.

## 5 Conclusion

Waves of SARS-CoV-2 infections suggest that coronavirus poses a sustainable threat to human life, even in contemporary times. Uncertainty will surround the epidemiological approach of relying solely on health certificates once peer-reviewed and validated data support claims that vaccination reduces SARS-CoV-2 transmission, which a health passport app can address. This pandemic might become endemic in due time because of weak viral mutations. The design of a health passport app for the pandemic will differ substantially from an app for an endemic because the restrictions during the endemic are relaxed, whereas they are stringent in the pandemic. We assume that the opinions discussed herein will be helpful to app developers in updating their versions. Similarly, health passports encourage many people to choose to be vaccinated instead of hesitating to be vaccinated, thereby contributing to herd immunity. Also, security should be the foundation of health passport development to give people confidence that their data is protected from misuse, falsification, and breaches of personal and health information privacy. This review article states current epidemiological obstacles in creating a pragmatic COVID-19 health passport and suggests possible solutions to address several of them. Researchers will need to conduct future research in several domains, depending on the evolution of COVID-19.

## Author contributions

RA: Data curation, Resources, Writing—original draft, Writing—review & editing. RS: Conceptualization, Formal analysis, Supervision, Writing—review & editing. JM: Conceptualization, Data curation, Formal analysis, Resources, Supervision, Writing—original draft, Writing—review & editing. AM: Data curation, Resources, Writing—original draft.
